# Efficacy, Safety, and Usability of Remifentanil as Premedication for INSURE in Preterm Neonates

**DOI:** 10.3390/children5050063

**Published:** 2018-05-22

**Authors:** Hadiyah Y. Audil, Sara Tse, Chad Pezzano, Amy Mitchell-van Steele, Joaquim M. B. Pinheiro

**Affiliations:** 1 Albany Medical College, Albany, NY 12208, USA; audilh@amc.edu; 2 Department of Pediatrics, Albany Medical College, MC-101, 47 New Scotland Avenue, Albany, NY 12208, USA; tses@amc.edu (S.T.); pezzanc@amc.edu (C.P.); mitchea@amc.edu (A.M.-v.S.)

**Keywords:** remifentanil, INSURE, intubation, surfactant, sedation, premedication

## Abstract

*Background*: We previously reported a 67% extubation failure with INSURE (Intubation, Surfactant, Extubation) using morphine as analgosedative premedication. Remifentanil, a rapid- and short-acting narcotic, might be ideal for INSURE, but efficacy and safety data for this indication are limited. *Objectives*: To assess whether remifentanil premedication increases extubation success rates compared with morphine, and to evaluate remifentanil’s safety and usability in a teaching hospital context. *Methods*: Retrospective review of remifentanil orders for premedication, at a large teaching hospital neonatal intensive care unit (NICU). We compared INSURE failure rates (needing invasive ventilation after INSURE) with prior morphine-associated rates. Additionally, we surveyed NICU staff to identify usability and logistic issues with remifentanil. *Results*: 73 remifentanil doses were administered to 62 neonates (mean 31.6 ± 3.8 weeks’ gestation). Extubation was successful in 88%, vs. 33% with morphine premedication (*p* < 0.001). Significant adverse events included chest wall rigidity (4%), one case of cardiopulmonary resuscitation (CPR) post-surfactant, naloxone reversal (5%), and notable transient desaturation (34%). Among 137 completed surveys, 57% indicated concerns, including delayed drug availability (median 1.1 h after order), rapid desaturations narrowing intubation timeframes and hindering trainee involvement, and difficulty with bag-mask ventilation after unsuccessful intubation attempts. Accordingly, 33% of ultimate intubators were attending neonatologists, versus 16% trainees. *Conclusions*: Remifentanil premedication was superior to morphine in allowing successful extubation, despite occasional chest wall rigidity and unfavorable conditions for trainees. We recommend direct supervision and INSURE protocols aimed at ensuring rapid intubation.

## 1. Introduction

Neonatal respiratory distress syndrome (RDS) occurs in nearly 50% of preterm infants born before 30 weeks’ gestation and it constitutes a significant cause of morbidity and mortality [1]. Prophylactic and early surfactant replacement therapy significantly decreases pulmonary complications and overall mortality [2,3]. Given the susceptibility of premature lungs to ventilation-induced lung injury, the current preferred management strategy for RDS emphasizes initial nasal continuous positive airway pressure (CPAP) with selective early surfactant replacement therapy in neonates requiring increasing oxygen supplementation [4,5,6]. Several approaches to delivering surfactant while minimizing invasive ventilation have been developed, beginning with the INSURE method (INtubation, SUrfactant, Extubation) [7], which is associated with a high success rate and reduced duration of respiratory support [8]. More recently, less invasive surfactant administration (LISA) or similar procedures have utilized an intratracheal catheter or feeding tube, or a laryngeal mask airway (LMA) [9,10]. Except for the LMA approach, all of these techniques involve laryngoscopy and tracheal cannulation or intubation.

Since multiple studies have demonstrated reduced adverse events and improved intubation conditions with use of premedication [11,12,13,14], the American Academy of Pediatrics (AAP) recommends using premedication for all non-emergent endotracheal intubations in neonates; however, it does not specifically address INSURE, in which rapid extubation is an additional goal [11]. While morphine is frequently used as premedication for neonatal intubation, its slow onset and long duration of action make it suboptimal, particularly for transient INSURE-type intubations [14,15]. It is also associated with significant adverse events, including hypotension and high rates of neurorespiratory depression and subsequent mechanical ventilation [16,17].

Recent small studies have explored the utilization of remifentanil, a synthetic opioid whose rapid onset and short duration of action might render it an ideal premedication for INSURE intubations [18,19,20]. Many adverse events associated with morphine premedication, notably prolonged respiratory depression, were not seen with remifentanil; however, identification of muscle rigidity in several neonates raised concerns about the drug’s safety profile [21,22]. Nevertheless, following our prior study that demonstrated high rates of morphine-associated extubation failure [23], our Center adopted remifentanil as the potentially best alternative to morphine [24], while recognizing the need for further study to evaluate remifentanil’s efficacy as INSURE premedication [25]. Herein, we review our experience with implementation of remifentanil premedication in our academic regional NICU, aiming to evaluate whether it improves INSURE success rates relative to morphine, and to assess the drug’s safety and usability.

## 2. Materials and Methods

Both components of this study were conducted in accordance with the Declaration of Helsinki, and the protocol was pre-approved by the Institutional Review Board of Albany Medical Center (protocol # 4586).

### 2.1. Retrospective Review

We performed a retrospective review of remifentanil orders to pharmacy during implementation of remifentanil premedication for INSURE during a 29-month period (January 2014 to May 2016). INSURE was performed on neonates with mild-moderate respiratory distress syndrome, who were receiving supported CPAP or nasal intermittent positive pressure ventilation (NIPPV) and FiO_2_ between 30% and 60% [23]. Remifentanil was reconstituted and diluted in pharmacy, with normal saline, to a concentration of 2 µg/mL, and infused at 2 μg/kg over 1 to 2 min, up to 5 min including subsequent slow flushing of the infusion tubing with saline; the dose was not titrated to effect, but infants were to be intubated once apneic, even if the infusion was still in progress. Atropine (0.01 mg/kg IV bolus) preceded remifentanil. Infants at <33 weeks’ gestation are routinely loaded with caffeine citrate prior to these procedures. In our 60-bed NICU, most intubations involving remifentanil were performed by staff credentialed to independently intubate neonates (fellows, nurse practitioners, physician assistants, respiratory therapists), under the supervision of an attending neonatologist; in some cases, residents undertook the initial attempt. The primary outcome was the rate of INSURE failure, defined as needing invasive ventilation one hour after beginning premedication. Secondary outcomes and process measures included the incidence of adverse events, interval between remifentanil order to pharmacy and administration, number of intubation attempts (defined by laryngoscope insertion), and the professional role of the intubating clinician(s).

### 2.2. Staff Survey

We also conducted an anonymous survey of NICU staff, housestaff, and pharmacists, using Qualtrics^®^ software (version July 2016, Qualtrics, LLC, Provo, UT, USA, www.qualtrics.com) to identify usability and logistical issues with remifentanil. Responders were asked to identify their medical care role, evaluate their experience and satisfaction with remifentanil use, and describe observations on administration, apparent efficacy, and adverse events (Appendix A).

### 2.3. Analyses

Descriptive statistical analyses were performed using Excel 2010 (Microsoft Corp., Redwood, WA, USA) and Stata 14 (StataCorp, College Station, TX, USA); the unit of analysis was each remifentanil dose, except for population demographics, which were analyzed at the patient level. Qualitative thematic analyses of free text responses were carried out using Excel string functions and Qualtrics tools, supplemented by manual review. We used chi-squared statistics to compare the rate of remifentanil-associated versus morphine-associated INSURE failure, designating statistical significance at *p* < 0.05. Our a priori power analysis had estimated that 50 remifentanil doses would be needed to detect a 20% absolute decrease in INSURE failure with a power of 80%. Finally, we used chi-squared statistics to evaluate the association between provider role and their opinions of remifentanil premedication from the survey data. 

## 3. Results

### 3.1. Retrospective Review

Eighty-three total remifentanil orders were sent to pharmacy, with three being duplicate, yielding 80 unique orders. From these orders, 73 doses of remifentanil were administered to 62 unique patients (mean gestational age 31.6 ± 3.8 (SD) weeks; mean birthweight 1.832 ± 0.886 kg); the remaining doses were not administered, likely due to delayed availability. Remifentanil administration was indicated specifically for INSURE in 81% of patients (65/80), and for other intubations in the remainder. The median age at INSURE was 19.5 h (interquartile limits 5.5 and 31.5 h).

**Figure 1:**
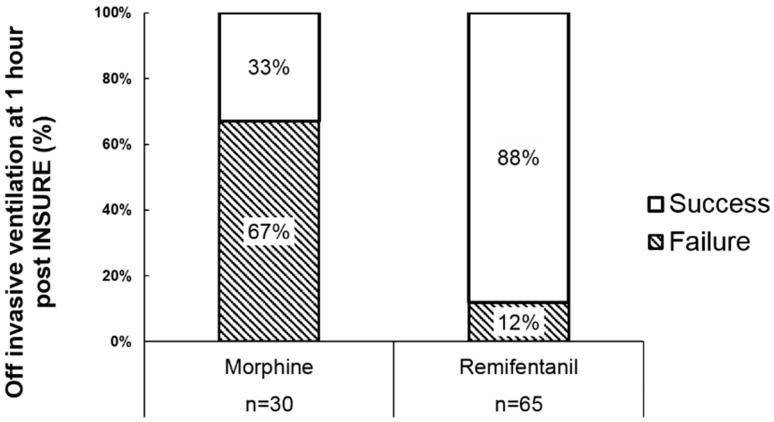
Failure rates with morphine versus remifentanil premedication. The proportion of extubation failure one hour after remifentanil premedication for INSURE (Intubation, Surfactant, Extubation) was significantly lower than that previously observed with morphine premedication (chi square test, *p* < 0.01).

At one hour post-remifentanil premedication, the extubation failure rate was 12% (8 of 65), significantly lower than the 67% failure rate following morphine premedication observed in a previous study of 30 patients at our center (Figure 1; *p* < 0.01) [23]. NIPPV and CPAP were utilized in 65% (42/65) and 23% (15/65) of patients, respectively, one hour after remifentanil administration.

Of the 73 remifentanil doses administered (including eight for non-INSURE intubations), documentation regarding adverse events was missing in 5%, but at least one adverse event was documented in 49%. Notable adverse events included significant desaturation (SpO_2_ < 70%) in 36%, and chest wall rigidity in 4% (Table 1). One infant was difficult to ventilate after surfactant administration and received chest compressions and naloxone, although the event was most likely related to surfactant obstruction of the airway rather than a remifentanil effect. Several other issues were recorded in 26% of neonates, including difficulty with bag-mask ventilation, laryngospasm or closed vocal cords, and delayed drug availability. 

The mean interval between time of remifentanil order to pharmacy and time of administration was 1.3 h, with 50% of cases documenting a delay of at least 1.1 h (interquartile limits 0.8 and 1.6 h). Although data regarding number of intubation attempts were missing in 34% of cases, the mean number of recorded attempts per intubation episode was 2.8. Only 14% of intubations were successful on the first attempt, and 12% required at least four intubation attempts (Figure S1). All neonates in our series were successfully intubated, except one who received surfactant through an LMA after five failed intubation attempts. Trainees (students, residents, and fellows) comprised 30% of first intubators but only 16% of final intubators; conversely, attending physicians were 8% of first intubators and 33% of final intubators (Figure S2).

### 3.2. Staff Survey

One hundred fifty-four respondents, 53% of which were nurses, completed 137 surveys (Table S1). In addition, 57% noted adverse events and/or logistical problems with remifentanil administration during the INSURE procedure, although significant differences in perception existed amongst various respondent roles (Figure S3; *p* = 0.03). A majority of nurse practitioners and physician assistants (NPs/PAs), neonatology fellows, and respiratory therapists indicated negative opinions of safety and efficacy of remifentanil usage; residents, attending physicians, and pharmacists had more ambivalent responses. Among adverse events reportedly observed by 61 respondents, the most commonly recalled were chest wall rigidity (36%), difficulty with bag mask ventilation (18%), and difficulty intubating (15%) (Figure S4). Respondents in the various roles also showed significant differences in perceived satisfaction with remifentanil premedication, with higher levels of satisfaction among fellows and attending physicians than among NPs/PAs and respiratory therapists (Figure S5; *p* < 0.01). Interestingly, there was no difference among providers in perceived effectiveness with remifentanil premedication (Figure S6; *p* = 0.14).

## 4. Discussion

This observational study substantially increases the reported evidence on remifentanil as INSURE premedication, while generalizing its use to a setting in which intubators may have wide-ranging experience in airway management. We found that while remifentanil was effective in inducing sedation, it provided a narrow therapeutic time window, hindering intubation for inexpert providers. 

The current preference for INSURE-type approaches for neonatal surfactant administration has created a need for rapid-onset analgosedative premedications that also allow immediate extubation, which, in our setting, typically occurs within about 10 min of intubation. Remifentanil and propofol share these characteristics, but they have only been evaluated in small pilot studies [26]. Remifentanil’s favorable hemodynamic effects and short half-life of about 5 min make it preferable to the more extensively-studied fentanyl, whose long half-life precludes rapid extubation. Welzing et al. successfully used remifentanil (2 µg/kg over 1 min) premedication in 21 preterm neonates undergoing INSURE [20]. Pereira e Silva et al. randomized 10 preterm neonates undergoing intubation for anesthesia to remifentanil 1 µg/kg over 1 min, along with midazolam, noting better intubation conditions than in the group randomized to morphine [19].

Having abandoned morphine premedication for INSURE due to the associated high extubation failure rates in a recent randomized trial [23], and desiring to continue using premedication for INSURE, we adopted a regime similar to Welzing’s infusing remifentanil at 2 µg/kg, albeit at a slower rate. To maximize safety, remifentanil powder was reconstituted, and the final dose prepared in the hospital pharmacy. The standard order set included naloxone to reverse potential chest wall rigidity. Whereas most intubations in our setting are performed by trained neonatal nurse practitioners, physician’s assistants, respiratory therapists, and neonatology fellows, some are done by less experienced residents in training under attending physician supervision. Our retrospective review of INSURE procedures under these conditions revealed a significantly improved rate of remifentanil-associated extubation failure compared with our prior morphine-associated rates. During both periods, guidelines to maintain infants intubated after surfactant included persistent apnea, severe retractions, or acutely increased oxygen requirement after surfactant; in the remifentanil era, extubation failure was attributable to suboptimal response to surfactant rather than persistent apnea. This supports evidence from pilot studies demonstrating the superiority of remifentanil over morphine [19] for neonatal intubation, and its ability to consistently permit extubation to non-invasive ventilatory support [20]. 

Ninety-nine percent of neonates in our series were successfully intubated. However, despite most initial intubators being experienced in airway management, first-attempt intubations had low success rates, suggesting post-remifentanil intubation conditions were suboptimal. Since these intubations were performed during routine clinical care, we did not formally score intubation conditions. Furthermore, without a control group utilizing a different analgosedative, we cannot discern whether remifentanil actually hindered the success of intubation. Nevertheless, the combined experiences of the authors and survey responses suggest that remifentanil rapidly induces hypoventilation and hypoxemia, and that any element of laryngospasm or chest wall rigidity may make intubation or bag-mask ventilation difficult. Some of remifentanil’s analgosedative effect wanes during bag-mask ventilation pre-intubation, and particularly during restabilization intervals between intubation attempts. Titration with additional small doses of remifentanil might prolong the sedative effect when desired, but we have not attempted this approach in our setting. Therefore, based on our review, we recommend that inexperienced intubators should not attempt intubations in which remifentanil is the only analgosedative. In addition, intubation should be attempted immediately upon the onset of hypoventilation and desaturation to minimize hypoxemia, even if the remifentanil infusion has not been completed.

Adverse effects were not directly captured through continuous physiologic monitoring but retrieved as documented in the medical records. Significant desaturation was most commonly noted, though this was rarely associated with bradycardia. Chest wall rigidity was noted in 4%, with these and one additional infant receiving naloxone. Among other adverse events recorded, difficulty with bag-mask ventilation, laryngospasm and closed vocal cords may also be attributable to remifentanil. Chest wall rigidity was reported by Choong et al. in 2 of 15 neonates (13%) given 3 μg/kg remifentanil over 60 s; the authors additionally noted difficulty in optimizing SpO_2_ before intubation, transient effects requiring redosing, and the need for open-label succinylcholine in 4 of the 15 neonates [22]. In a recent report of remifentanil premedication for INSURE, de Kort et al. infused remifentanil 2 µg/kg over 30 s and observed chest wall rigidity in 43% of 14 preterm neonates, while obtaining adequate sedation in only 14% [27]. The manufacturer notes that bolus doses of >1 µg/kg administered over 30–60 s may cause chest wall rigidity. Concurrent use of sedatives [19] or muscle relaxants may diminish this effect, but neither is desirable in INSURE. In contrast, Welzing et al. did not observe chest wall rigidity using a dosing procedure similar to ours, although their sample size was small [20]. About one third of our survey responders indicated that chest wall rigidity was the most significant adverse effect, followed by difficulty in bag-mask ventilation and intubation. Because multiple caregivers witnessed a given intubation, this likely represents an augmented recall of such events, rather than actual incidence. Nonetheless, the staff members who most commonly performed INSURE procedure intubations (nurse practitioners, physician’s assistants, fellows, respiratory therapists) had a more positive perception of the effectiveness of remifentanil, despite reportedly witnessing frequent adverse effects. Overall, attending and fellow physicians, who comprised a minority of respondents and intubators, were at least somewhat satisfied with remifentanil premedication, whereas dissatisfaction predominated among staff members in other roles. This suggests it is challenging to implement remifentanil premedication in a large teaching hospital NICU where numerous individuals perform intubations, sometimes infrequently. Correspondingly, Welzing et al. attributed failed intubations in their study to residents in training, despite good or excellent intubation conditions [20].

Because remifentanil requires reconstitution and the 500-fold dilution is complicated, the prescribed dose was prepared in the hospital pharmacy to maximize safety. The short shelf life of the final solution precludes routine preparation in advance. These factors, and the distance between pharmacy and NICU, cause a substantial time lag between drug prescription and administration, which may delay surfactant administration to patients and disrupt NICU staff workflows. These problems, as well as staff time, drug wastage, and possibly the predictability of dose administration, would be improved if a neonatal formulation were available. 

Finally, remifentanil premedication in neonates with RDS creates a narrow therapeutic time window of adequate sedation with physiologic stability, which is unpropitious to inexperienced trainees. Leone et al. [28] noted that, in 2002, pediatric residents averaged 12 newborn intubation attempts over three years of training. Our experience has led us to avoid having inexperienced trainees attempt intubations for INSURE, and to recommend second intubation attempts be immediately performed by the most experienced intubator. Given the increased frequency of INSURE and the advent of the LMA use [23], there is concern, also noted by some survey respondents, that the intubation competence of pediatric residents may be further delayed.

In the context of the deliberate change in premedication practice in our NICU, there was no equipoise for a prospective, randomized trial, nor was this deemed feasible within a reasonable time horizon. Consequently, our pragmatic observational study has several limitations. The quality of data obtained by retrospective review is neither uniform nor always complete, despite the use of procedure documentation forms; however, the retrospective study could not have biased or limited the clinical documentation. It would have been helpful to score intubation conditions, but we do not do this routinely; furthermore, intubation conditions change rapidly during remifentanil administration, and a single value cannot describe the status at initial laryngoscopy and subsequent attempts. Our staff survey likely has inherent responder biases, but the impact of these was minimized by focusing on qualitative rather than quantitative analyses. Additionally, the exact rate of remifentanil infusion was unmeasured; however, this is impossible to estimate precisely because some of the drug remains in the intravenous tubing and is pushed in by the saline flush. This problem might be lessened by a neonatal formulation of remifentanil, either consistently prepared across pharmacies or commercially available. Finally, our findings reflect practical use in a setting where remifentanil is prepared in a central pharmacy, and where multiple clinicians of varying experience perform INSURE procedures; they may not be generalizable with different methods of preparing the remifentanil solution, or under stricter protocol conditions practiced in prospective studies.

We considered multiple alternative drugs as potential premedication for INSURE, before choosing remifentanil. Of those, propofol seemed to be another reasonable option [26], although it has no analgesic properties. Furthermore, we were dissuaded by reports of varying degrees of hypotension, which still occurred in 14% of neonates in a recent study by Descamps et al. on premedication for less invasive surfactant administration, despite starting propofol titration at 0.5 mg/kg and adding nalbuphine in selected cases [29].

## 5. Conclusions

In conclusion, our study demonstrates that remifentanil has the potential to promote successful INSURE procedures, while also highlighting adverse effects. Given the need for updated labeling on this and most other drugs used in premature infants [30], our practice experience should be helpful to clinicians intending to design prospective studies, and to NICU planning to implement remifentanil use for INSURE in routine clinical care.

## Figures and Tables

**Table 1:** Documented adverse events seen with remifentanil premedication.

Adverse Event Documented	n/N	Percent of Valid Data
Missing all documentation	4/73	5%
Desaturation	26	36%
Bradycardia	6	8%
Trauma	3	4%
Pneumothorax within 2 h	3	4%
Pneumothorax beyond 2 h	2	3%
Chest wall rigidity	3	4%
CPR (post-surfactant)	1	1%
Apnea	6	8%
Medications/naloxone	4	5%
Other adverse event	19	26%
Any adverse event	36	49%

CPR, cardiopulmonary resuscitation.

## Supplementary Materials

The following are available online at http://www.mdpi.com/2227-9067/5/5/63/s1; Table S1: Survey respondents’ professional roles; Figure S1: Distribution of attempts per intubation; Figure S2: Intubator role for first and final intubation attempts; Figure S3: Perceived adverse effects and/or logistical problems with remifentanil administration amongst various respondent roles; Figure S4: Observed adverse events, according to survey respondents; Figure S5: Perceived satisfaction with remifentanil premedication amongst various respondent roles; Figure S6: Perceived effectiveness of remifentanil premedication amongst various respondent roles. A deidentified data file (Summary+Remifentanil_Data_166+DEIDENTIFIED.xlxs), in Excel format, is available to readers.

## Appendix A

Staff survey on remifentanil for INSURE premedication.

**REMIFENTANIL FOR INSURE PREMEDICATION SURVEY**

*This 1 to 3 min, IRB-approved survey aims to assess user experiences with remifentanil as premedication for intubation in NICU. Please answer questions 1–2 even if you have not witnessed remifentanil use. All responses are anonymous. **Thank you!***

**1. Approximately how many cases have you been involved in, *either as observer or as care provider*, for using remifentanil as induction premedication to INSURE (Intubation, Surfactant, Rapid Extubation)?**
  (a) 0	(b) 1–2	(c) 3+
**2. What is your work title or function?**
  (a) Resident	(e) Attending
  (b) NP/PA	(f) RN
  (c) NP or PA student	(g) Respiratory therapist
  (d) Fellow	(h) Pharmacist
**3. What role(s) have you played in the INSURE procedures? (*check all that apply*)**
  (a) Intubator	(c) Supervisor	(e) None of these
  (b) Other direct care provider	(d) Observer only	
**4. Do you feel remifentanil premedication provides *effective* analgesia/sedation for neonatal intubation?**
  (a) Yes
  (b) Maybe
  (c) No—If no explain: ____________________________________________________________________
  ________________________________________________________________________________________
**5. Were there any adverse effects or logistical problems with remifentanil administration during the INSURE procedures?**
  (a) No
  (b) Yes—if yes explain: ___________________________________________________________________
  ________________________________________________________________________________________
**6. What is your overall satisfaction with remifentanil as an induction premedication to INSURE?**
  (a) Very satisfied
  (b) Somewhat satisfied
  (c) Neither satisfied nor dissatisfied
  (d) Somewhat dissatisfied
  (e) Very dissatisfied
**7. Based on your experiences please provide any additional feedback or comments regarding remifentanil as an induction premedication to INSURE (both positive and negative).**
  ________________________________________________________________________________________________________________________________________________________________________________________________________________________________________________________________________________________________________________________________________________________________
