# Supplementary material for: Efficacy, Safety, and Usability of Remifentanil as Premedication for INSURE in Preterm Neonates
Source: Children (Basel). 2018 May 22;5(5):63. doi: 10.3390/children5050063 (PMC5977045; doi:10.3390/children5050063)
Supplement: Supplementary file 1 [file children-05-00063-s001.zip › children-302284 suplementary 2.pdf]

Supplemental information, online only

1. Table S1. Survey respondents’ professional roles
2. Figure S1. Distribution of attempts per intubation
3. Figure S2. Intubator role for first and final intubation attempts
4. Figure S3. Perceived adverse effects and/or logistical problems with remifentanil administration amongst various respondent roles
5. Figure S4. Observed adverse events, according to survey respondents
6. Figure S5. Perceived satisfaction with remifentanil premedication amongst various respondent roles
7. Figure S6. Perceived effectiveness of remifentanil premedication amongst various respondent roles

Table S1. Survey respondents' professional roles

**Respondents' professional role**	**Count**	**%**
Resident	22	14.3%
Nurse practitioner / physician's assistant (NP/PA)	14	9.1%
NP/PA student	1	0.7%
Fellow	3	2.0%
Attending	12	7.8%
Registered nurse (RN)	82	53.3%
Respiratory therapist	12	7.8%
Pharmacist	8	5.2%
**Total**	**154**	**100.0%**

Figure S1. Distribution of attempts per intubation

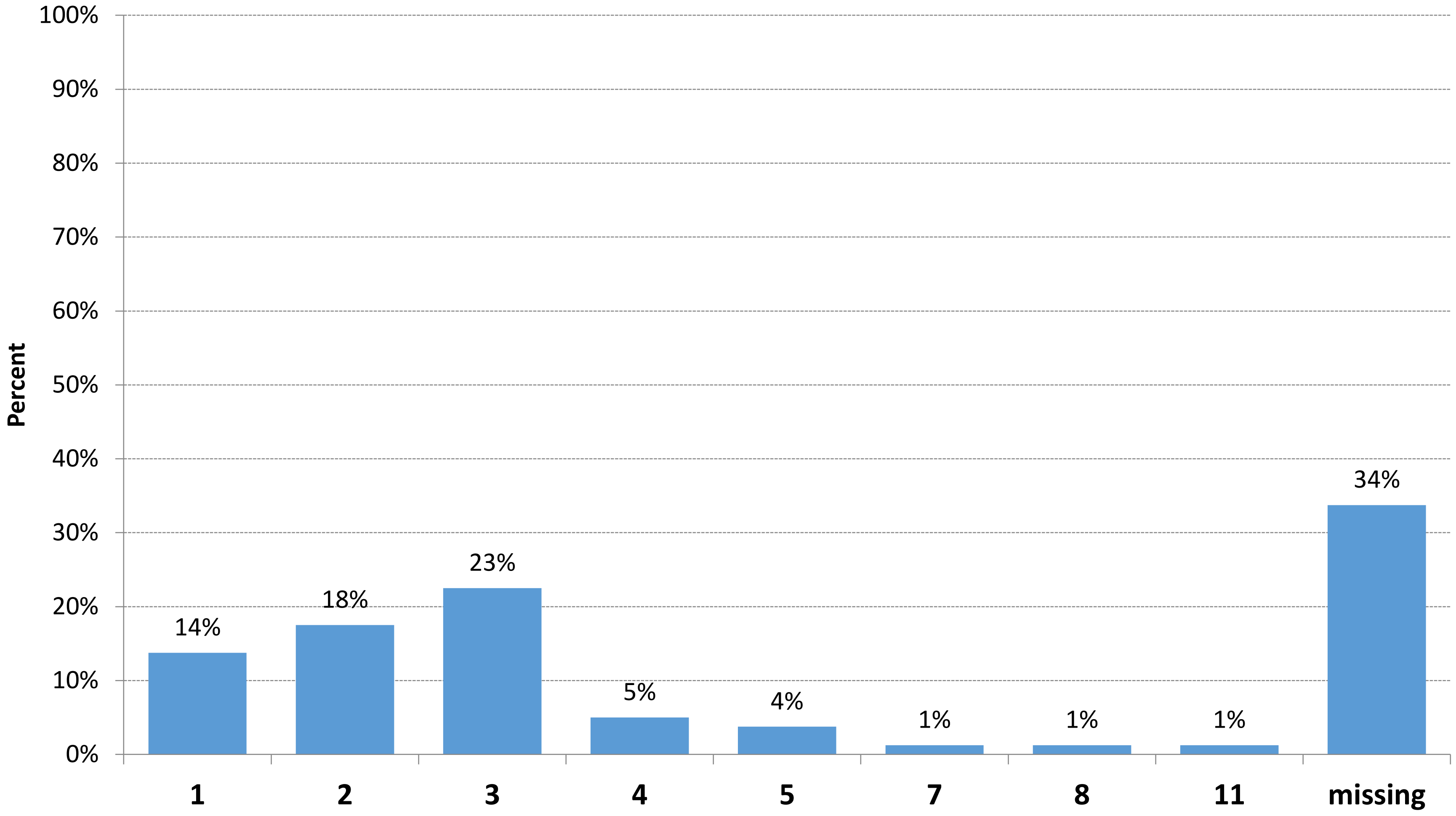

Figure S2. Intubator role for first and final intubation attempts

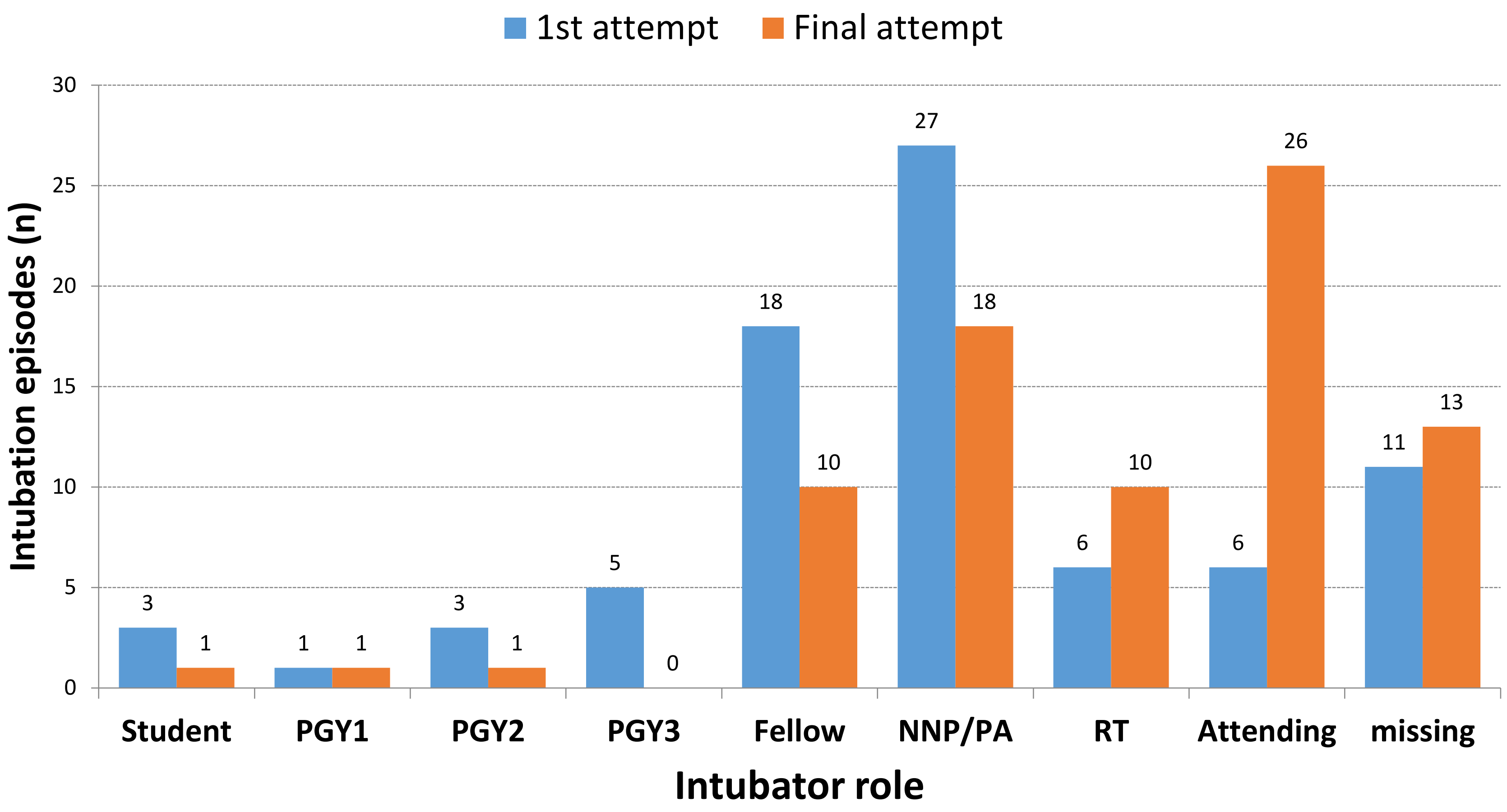

PGY denotes postgraduate year of residency training; NNP/PA, neonatal nurse practitioner or physician’s assistant; RT, respiratory therapist

Figure S3. Perceived adverse effects and/or logistical problems with remifentanil administration amongst various respondent roles

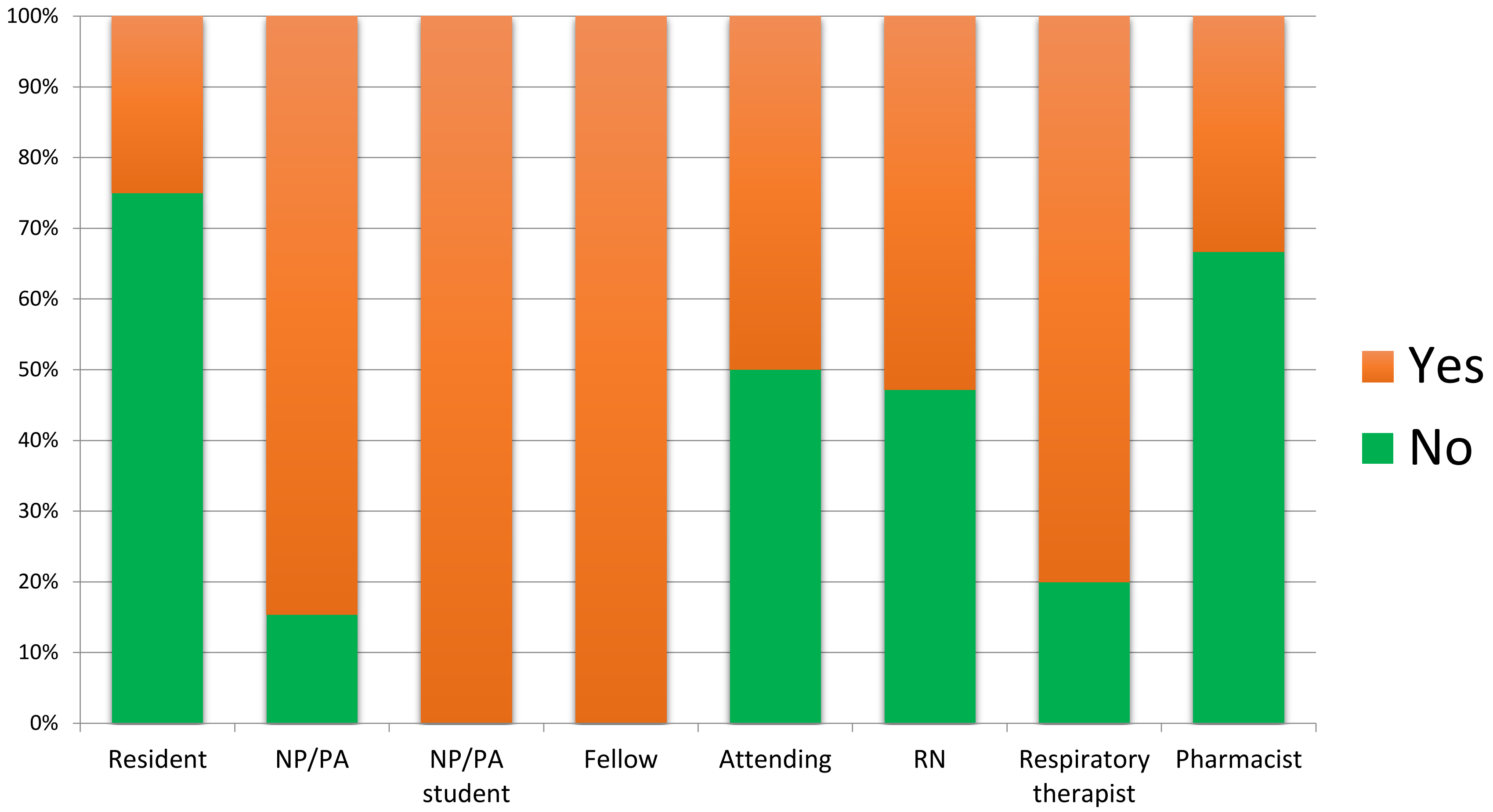

NP/PA, nurse practitioner or physician's assistant; RN, registered nurse

There were significant differences in perception of adverse events or drug availability among the various respondent roles (chi square, p=0.03)

Figure S4. Observed adverse events, according to survey respondents

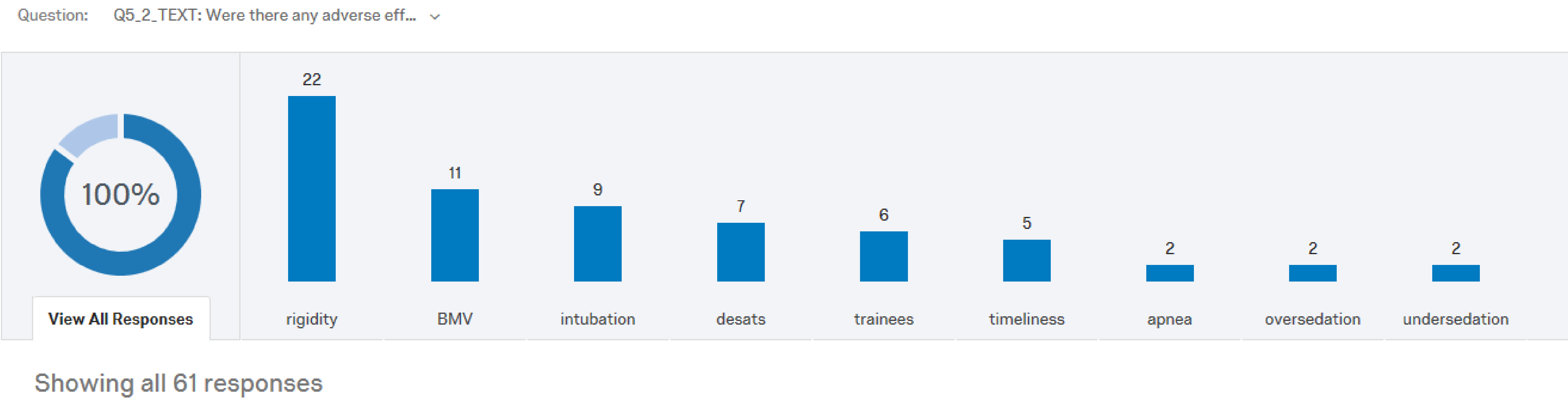

BMV, bag-mask ventilation; desats, desaturations

Figure S5. Perceived satisfaction with remifentanil premedication amongst various respondent roles

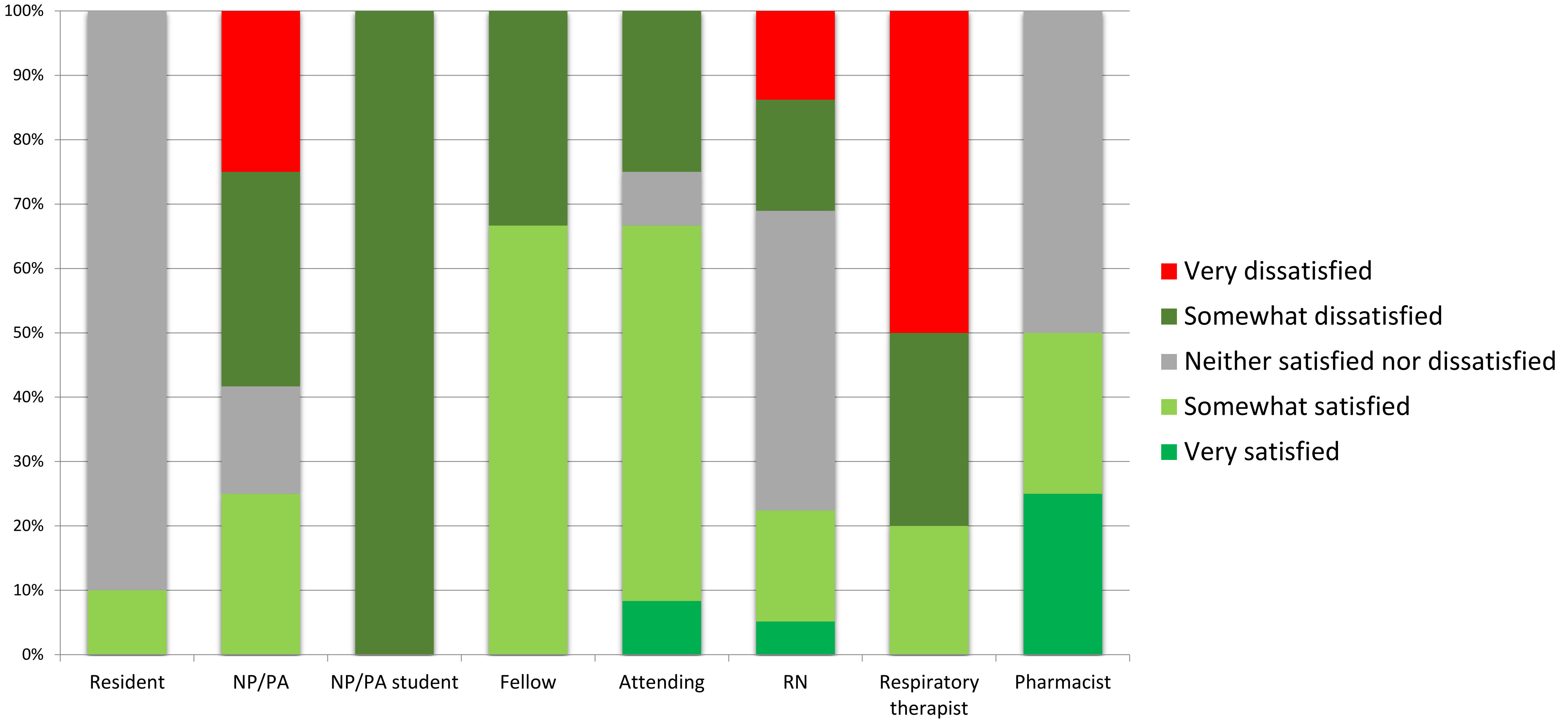

NP/PA, nurse practitioner or physician's assistant; RN, registered nurse

There were significant differences in reported satisfaction with remifentanil premedication among the various respondent roles (chi square, $p<0.01$)

Figure S6. Perceived effectiveness of remifentanil premedication amongst various respondent roles

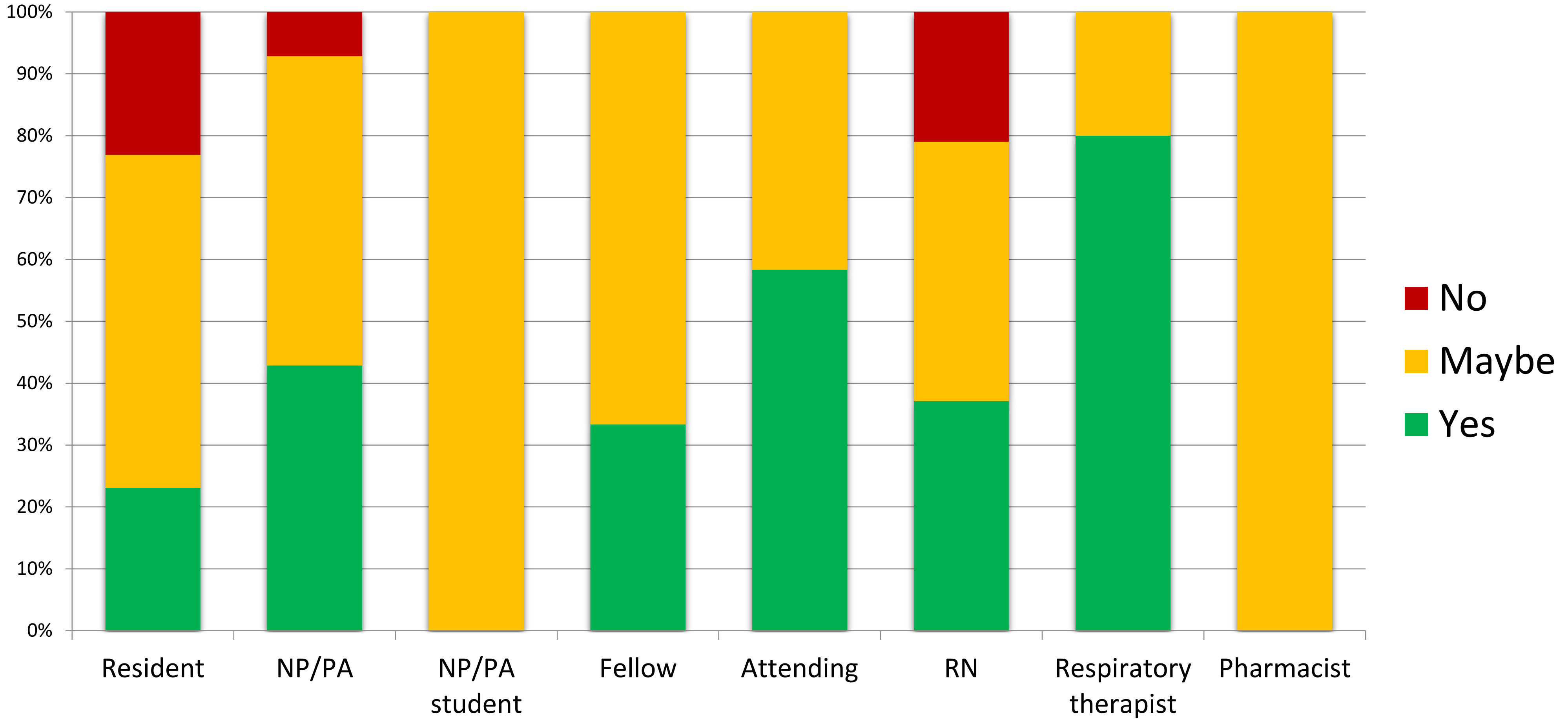

NP/PA, nurse practitioner or physician's assistant; RN, registered nurse
There were no significant differences in perceived effectiveness of remifentanil premedication among the various respondent roles (chi square, p=0.24)
